# How journal editors can support women in chemistry

**DOI:** 10.1038/s42004-022-00638-y

**Published:** 2022-03-08

## Abstract

This International Women’s Day, the editors of *Communications Chemistry* reflect on the responsibilities that journal editors have towards promoting gender equity in STEM, and outline some of the ways in which editors can show up for women in chemistry.

## Gender disparity

Articles promoting gender equity often begin by pointing to data that showcase relevant gender disparities. For many women in STEM, the lived experience is unfortunately sufficient evidence of the problem. Nonetheless, understanding the scale of gender disparity is important, and we now have access to plenty of data demonstrating that fewer women enter and (crucially) stay in STEM education or STEM professions than men. Focusing on academia, 2016 data from the UNESCO Institute for Statistics found that only 29% of the world’s STEM researchers were women. A 2018 report published by the Royal Society of Chemistry highlighted that the percentage of women in academia in the chemical sciences in the UK falls from 39% at the PhD student level to 9% at professor level. A 2021 STEM Equity Monitor report from the Australian government found that women made up less than 25% of students studying STEM subjects in Australia in 2019.

An important aspect that these statistics also don’t show is ‘which women’: gender-related data are typically not disaggregated by other identities such as sexual orientation, ethnicity, or disability, and do not usually include data on minority genders, thereby hiding the barriers that women at the intersection(s) of minoritized identities face.

So we ask ourselves, how can we, as journal editors, support women researchers—from all communities—and help to create a more equitable environment?

## Affording women with equitable opportunities

Affording women with equitable opportunities is surely an important start. When recruiting Editorial Board Members, or when we invite researchers to peer review a paper, to write a Review article or opinion piece, or to give a plenary lecture, for example, we must ensure that women are invited to the same playing field as men. In practice, it can be all too easy for a busy journal editor to approach the ample visible male academics in any given field, contributing to a vicious cycle where easy-to-find, prominent men are approached for opportunities, perpetuating their comparably greater visibility. Searching further afield for the often (but not always) less visible, established or up-and-coming women researchers can certainly take longer, and in some fields more so than in others, but it is an inherent part of an editor’s role and responsibility within the community.

We are also aware that many women, particularly those who are at the intersection of marginalized identities, already carry out the majority of academic service—taking comparatively more time and resources (both physical and mental ones) from their research than their white male colleagues^1,2^. We are mindful of this and certainly do not wish to over-burden the same few women. Ongoing efforts to diversify our reviewer, author, and speaker pools should help to distribute opportunities as well as commitments among scientists.

Beyond ensuring a diverse line-up of speakers, organizing conferences with mindfulness towards those with caring responsibilities (typically more prevalent for female academics than male academics) can also help to support women in STEM. For example, scheduling conference and travel days during the working week, and providing child care options, can help facilitate attendance.

When considering what research to promote, through Research Highlights, journal covers, marketing campaigns, collections, or on social media, editors should be mindful of the gender of those whose voices they choose to amplify. Travel or training grant opportunities, reviewer recognition awards and professional editor positions could also be awarded to as many women as they are men.

## Minimizing bias

The question of bias in publishing is an important one. In 2019, the Royal Society of Chemistry published a report—Is Publishing in the Chemical Sciences Gender Biased—that disclosed that female corresponding authors are less likely to have their papers accepted in a Royal Society of Chemistry journal than men. They attributed this to the combined effect of subtle biases that occur at every step of the peer review and publishing process. Of course, this phenomenon is extremely unlikely to be unique to RSC journals, and it is paramount that journal editors and peer reviewers learn to be conscious of their unconscious biases.

Manuscripts should be assessed on the basis of their scientific merit only. Decisions to publish papers should never rest on the identities of those on the author list. To help reach that goal, journal editors should receive training on minimizing their unconscious bias. While women are not exempt from experiencing bias against women, having a diverse and gender-balanced Editorial Board and peer reviewers can also help to minimize the problem. Publishers could also consider putting in place peer review models that eliminate opportunity for bias, such as triple anonymised peer review, where neither the reviewers nor the editors are privy to information about author identities.

Nature Portfolio journals do not currently possess data on the gender identities of those who submit to or get published in our journals. As journal editors we must, however, have the tools to measure any gaps in acceptance rates between genders in order to identify issues and inform our strategy. Springer Nature has signed the Royal Society of Chemistry’s joint commitment for action on inclusion and diversity in publishing, and has thereby committed to gathering and analysing this data.

## Being part of the conversation and celebrating our colleagues

Beyond the science, journals are well placed to offer a home for the discussion of equity issues that communities face^3,4^. Scientists in marginalized communities can be celebrated through articles such as researcher profiles or Q&As^5,6^. Journals can also support (financially and non-financially) meetings designed to celebrate minoritised groups, and publish the take-home messages from such meetings^7,8^.

Today, we at *Communications Chemistry* are pleased to launch a collection Celebrating Women in Chemistry, showcasing excellent research from women corresponding authors who have recently published in the journal.

We fully acknowledge that we have work to do when it comes to practicing all of the above, and we look forward to continuing to explore ways in which we can show up for women in chemistry. If you have feedback or suggestions for us, we would be most delighted to hear from you.
